# Risk-guided cardioprotection with carvedilol in patients with breast cancer (CCT guide): a phase 1 randomized clinical trial

**DOI:** 10.1007/s10549-025-07636-3

**Published:** 2025-04-02

**Authors:** Wonyoung Jung, Rebecca A. Hubbard, Amanda M. Smith, Kyunga Ko, Anran Huang, Jessica Wang, Jordan M. Isaacs, Liyong Zhang, Peter P. Liu, Zhen Chen, Payal D. Shah, David Mintzer, Saveri Bhattacharya, Hayley M. Knollman, Amy S. Clark, Daniel Koropeckyj-Cox, Melissa Messinger, Nicholas S. Wilcox, Congying Xia, Vivek Narayan, Jenica N. Upshaw, Saro H. Armenian, Bonnie Ky

**Affiliations:** 1 Division of Cardiology, Department of Medicine, Perelman School of Medicine, University of Pennsylvania, Philadelphia, PA USA; 2 Department of Biostatistics, Brown University School of Public Health, Providence, RI USA; 3 Division of Hematology and Oncology, Department of Medicine, Perelman School of Medicine, University of Pennsylvania, Philadelphia, PA USA; 4 University of Ottawa Heart Institute, University of Ottawa, Ottawa, ON Canada; 5 Division of Cardiology, Tufts Medical Center, Boston, MA USA; 6 Department of Pediatrics and Department of Population Sciences, City of Hope Comprehensive Cancer Center, Duarte, CA USA; 7 Abramson Cancer Center, Penn Center for Quantitative Echocardiography, Perelman School of Medicine at the University of Pennsylvania, 3400 Civic Center Boulevard, Philadelphia, PA 19104 USA; 8 Department of Biostatistics, Epidemiology and Informatics, Perelman School of Medicine, University of Pennsylvania, Philadelphia, PA USA

**Keywords:** Anthracyclines, Breast cancer, Cardiotoxicity, Carvedilol, Phase 1, Risk-guided

## Abstract

**Purpose:**

Breast cancer treatment results in increased cardiotoxicity risk; a risk-guided approach to cardioprotection has not been fully tested.

**Methods:**

This single-center, randomized Phase I trial enrolled patients with Stage I–III breast cancer who planned to receive anthracycline and/or trastuzumab therapy. An internally validated cardiotoxicity risk score classified participants as low or elevated risk. Elevated risk participants were randomized to receive open-label carvedilol or usual care for 12 months, beginning at cancer therapy initiation. Study visits occurred at baseline, 3, 6, 9, 12, and 24 months. Primary outcomes included feasibility, safety, and tolerability. Exploratory outcomes included echocardiography, biologic, and patient-reported measures.

**Results:**

Of the 166 eligible patients approached, 68 (41%) agreed to participate and ultimately enrolled. Among these participants (median age 52, 35% Black), 49 were classified as low and 19 elevated risk. Within the elevated risk group, 13 were randomized to carvedilol and 6 usual care. For those randomized to carvedilol, the median maximum dose was 6.25 mg twice daily, with 93% adherence. Adverse events of interest (grade 3 + bradycardia, hypotension, or fatigue) occurred in 9% with carvedilol, 13% in usual care, and 4% in low risk groups. One (1.5%) low risk participant experienced cardiac dysfunction. There were no substantial differences in secondary outcomes across groups. The participant withdrawal rate was 7%.

**Conclusions:**

This Phase 1 trial demonstrates that a risk-guided strategy can be applied to patients with active cancer. However, additional strategies are necessary to optimize the design and execution of non-treatment intervention trials in patients with active cancer.

**Trial registration:**

NCT04023110.

**Supplementary Information:**

The online version contains supplementary material available at 10.1007/s10549-025-07636-3.

## Introduction

Advances in cancer detection and treatment have significantly improved survival rates for patients with breast cancer [1]. However, the management of cancer treatment-related adverse events (AEs), particularly cardiotoxicity, remains an important challenge [2]. Cardiac dysfunction is one of the more significant AEs associated with commonly used systemic therapies in breast cancer, such as anthracyclines and trastuzumab [3, 4]. Measures to mitigate the risk of cardiac dysfunction include decreased anthracycline use, limited use of combination cardiotoxic therapies, and implementation of cardioprotective strategies [5].

Nevertheless, standardized approaches for cardioprotection remain limited. Much of the science in cardio-oncology has focused on the use of neurohormonal therapy in relatively unselected populations receiving anthracyclines [6]. Carvedilol is part of guideline-directed medical therapy in heart failure with reduced ejection fraction and has been shown to reduce heart failure hospitalizations, mortality, and adverse cardiac remodeling [7–9]. However, its role in cardio-oncology as a cardioprotective, prophylactic measure has yielded mixed results, with modest benefits often attributed to the inclusion of patients at low risk for cardiac dysfunction [10–12]. We hypothesized that a risk-guided strategy could be a safe, feasible, and efficacious approach to cardioprotection, focusing on higher risk patients who might derive greater benefit from pharmacologic intervention.

CCT Guide was a Phase 1 randomized clinical trial of risk-guided cardioprotection using open-label carvedilol in patients with nonmetastatic breast cancer initiating anthracyclines and/or trastuzumab therapy. The study was designed to test questions related to the feasibility, safety, and tolerability of a risk-guided approach. We also explored the efficacy of such an approach through the detailed assessment of secondary outcomes, including echocardiography-derived measures of cardiac function and remodeling, biomarkers of injury and stress, and patient-reported outcomes (PROs).

## Methods

### Study design and participants

In this single-center, prospective, randomized, open-label Phase I clinical trial (NCT04023110), women diagnosed with nonmetastatic breast cancer undergoing anthracycline and/or trastuzumab therapy were enrolled. Eligibility criteria included women aged ≥ 18 years who were planned for adjuvant or neoadjuvant therapy with anthracyclines and/or trastuzumab. Exclusion criteria included stage IV breast cancer at enrollment, pregnancy or breastfeeding, contraindications to carvedilol, current beta-blocker therapy, or inability to provide informed consent. Detailed eligibility criteria for the study population are shown in Supplemental Table 1.

**Table 1:** General characteristics of study population

	Total study population (*n* = 68)	Low risk, nonrandomized (*n* = 49)	Elevated risk, usual care (*n* = 6)	Elevated risk, carvedilol (*n* = 13)
Age	52 (42, 61)	52 (41, 60)	51 (47, 56)	47 (47, 62)
Race				
White	37 (54.4%)	29 (59%)	1 (17%)	7 (54%)
Black/AA	24 (35.3%)	17 (35%)	4 (67%)	3 (23%)
Non-AA/Non-White	7 (10.3%)	3 (6%)	1 (17%)	3 (23%)
Ethnicity				
Hispanic	1 (1%)	0 (0%)	1 (17%)	0 (0%)
Non-Hispanic	67 (99%)	49 (100%)	5 (83%)	13(100%)
Breast cancer laterality				
Left	33 (49%)	24 (49%)	4 (67%)	5 (38%)
Right	30 (44%)	22 (45%)	1 (17%)	7 (54%)
Bilateral	4 (6%)	2 (4%)	1 (17%)	1 (8%)
Node only	1 (2%)	1 (2%)	0 (0%)	0 (0%)
Cancer stage				
I	15 (22%)	9 (18%)	2 (33%)	4 (31%)
II	37 (54%)	31 (63%)	2 (33%)	4 (31%)
III	16 (24%)	9 (18%)	2 (33%)	5 (38%)
Molecular subtype				
HR- HER2 +	5 (7%)	3 (6%)	1 (17%)	1 (8%)
HR + HER2−	29 (43%)	21 (43%)	2 (33%)	6 (46%)
TNBC	16 (24%)	8 (16%)	3 (50%)	5 (38%)
TPBC	18 (26%)	17 (35%)	0 (0%)	1 (8%)
Cancer therapy				
Cancer treatment regimen				
Dox	40 (59%)	26 (52%)	4 (67%)	10 (77%)
Tras	26 (38%)	23 (46%)	1 (17%)	2 (15%)
Dox + Tras	2 (3%)	0 (0%)	1 (17%)	1 (8%)
Pembrolizumab, Yes	7 (10%)	4 (8%)	1 (17%)	2 (15%)
Radiation therapy, Yes	51 (76%)	37 (76%)	4 (67%)	10 (77%)
Left-sided radiation, Yes	27 (40%)	20 (41%)	3 (50%)	4 (31%)
Cardiovascular history/risk factors				
Current or prior smoking	22 (32%)	14 (29%)	2 (33%)	6 (46%)
History of hypertension	14 (21%)	10 (20%)	2 (33%)	2 (15%)
History of diabetes mellitus	5 (7%)	2 (4%)	1 (17%)	2 (15%)
History of hyperlipidemia	17 (25%)	14 (29%)	2 (33%)	1 (8%)
History of coronary artery disease	8 (12%)	7 (14%)	1 (17%)	0 (0%)
History of heart failure	0 (0%)	0 (0%)	0 (0%)	0 (0%)
History of arrhythmia	4 (6%)	4 (8%)	0 (0%)	0 (0%)
Cardiovascular medications				
ACEi or ARB use	8 (12%)	6 (12%)	1 (17%)	1 (8%)
Statin use	8 (12%)	6 (12%)	1 (17%)	1 (8%)
Clinical or laboratory measures				
Systolic blood pressure (mmHg)	125 (116, 138)	125 (117, 136)	121 (114, 138)	127 (105, 149)
Diastolic blood pressure (mmHg)	77 (73, 83)	77 (72, 82)	82 (77, 84)	77 (74, 85)
Resting heart rate (bpm)	76 (68, 83)	76 (66, 85)	86 (81, 95)	72 (68, 78)
Body mass index (kg/m^2^)	28.0 (23.3, 34.8)	28.7 (23.8, 36.1)	31.4 (27.9, 33.1)	25.2 (22.1, 30.2)
LVEF,^a^ % (Biplane)	61.2 (59.3, 64.8)	61.9 (60.0, 65.0)	59.6 (57.6, 59.9)	61.1 (58.2, 64.8)
hs-TnT, ng/L	6.0 (4.2, 8.0)	5.7 (4.1, 8.0)	6.2 (6.1, 8.6)	7.1 (5.1, 7.9)
NT-proBNP, ng/L	88.4 (37.1, 133.1)	98.4 (35.5, 161.6)	43.9 (42.5, 44.8)	88.4 (71.2, 109.4)

The study population is presented as intention-to-treat, with data shown as median (Q1, Q3) or number (%)

*AA* African-American; *HR* hormone receptor; *HER2* human epidermal growth factor receptor 2; *TNBC* triple-negative breast cancer; *TPBC* triple-positive breast cancer; *Dox* doxorubicin; Tras, trastuzumab; *ACEi* Angiotensin-Converting Enzyme Inhibitors; *ARB* Angiotensin II Receptor Blockers; *LVEF* left ventricular ejection fraction

^a^Quantitated EF was used. If quantitated values were unable to be analyzed, clinical LVEF was reported

The study was approved by the University of Pennsylvania’s Institutional Review Board and conformed to the Declaration of Helsinki standards. All participants provided written informed consent.

### Cardiotoxicity risk score

Our cardiotoxicity risk score was derived from the Cardiotoxicity of Cancer Therapy (CCT, NCT01173341) cohort study, an ongoing prospective longitudinal study of patients aged ≥ 18 years old with breast cancer treated with doxorubicin and/or trastuzumab at the University of Pennsylvania Health System.

A Least Absolute Shrinkage and Selection Operator (LASSO)-regularized Cox proportional hazards model was developed to identify key predictors associated with the time to onset of cardiac dysfunction, defined as a decline in left ventricular ejection fraction (LVEF) ≥ 10% from baseline to < 50%. Potential predictors included age, race (White, Black/African American, or Other), type of systemic cancer therapy (anthracyclines, trastuzumab or the combination), radiation therapy, smoking status, systolic blood pressure (SBP), body mass index (BMI), presence of diabetes, hypertension, and hyperlipidemia, and LVEF at baseline. Age, SBP, BMI, and LVEF were treated as continuous variables, while others were categorized. We employed tenfold cross-validation within each 90% training sample to fine-tune the LASSO penalty parameter without employing nested cross-validation and calculated the area under the receiver operating characteristic curve (AUC) at 1 year in the 10% validation sample. This cross-validation process was iterated across all 10 subsets, and an average AUC was derived. The final cardiotoxicity risk score was calculated using Cox proportional hazards regression with race, SBP, baseline LVEF, systemic cancer therapy, smoking status, diabetes, and hypertension as selected predictors. The model demonstrated an average cross-validated AUC of 0.72. Calibration was assessed using the Gronnesby and Borgan test.

A risk score threshold of −4.75, which is equivalent to a 4.5% risk of cardiac dysfunction within a year, was set to prioritize sensitivity, classifying 56% of participants as elevated risk. At this threshold, the model achieved a sensitivity of 81%, specificity of 56%, negative predictive value of 86%, and a positive predictive value of 12% for predicting cardiac dysfunction at one year in the development cohort. Lack of calibration was not significant (*p* = 0.07). Additional details of the risk prediction model are described in the Supplementary Material.

### Stratification and randomization

**Fig. 1:**
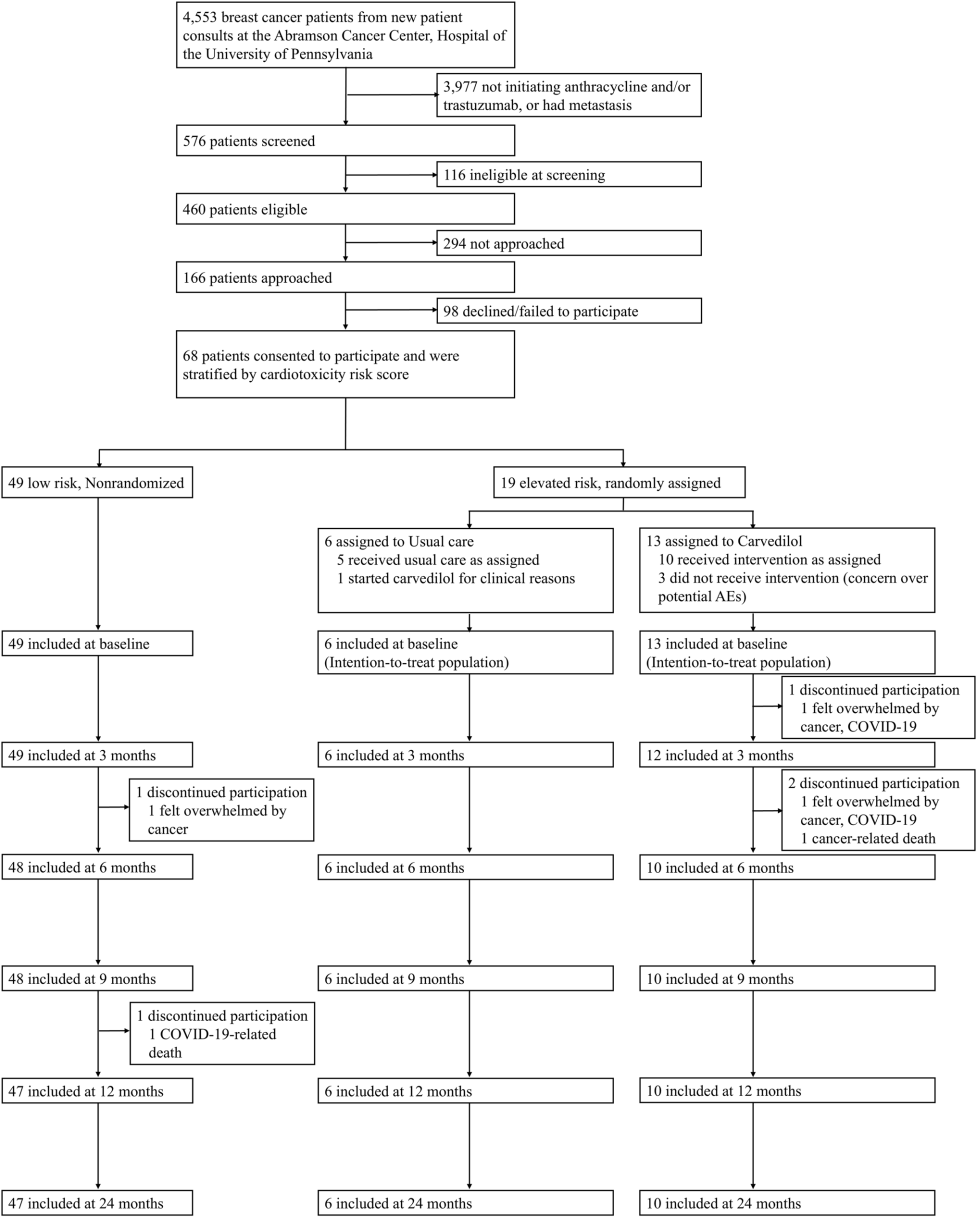
CONSORT diagram. This figure presents a flowchart detailing the enrollment process and participant progression throughout the trial. Four participants were screen failures post-consent due to taking a beta-blocker at the time of consent (*n* = 1), changes in the cancer treatment plan (*n* = 2), and a lack of a pregnancy test (*n* = 1)

Participants identified as being at elevated risk for cardiac dysfunction were randomized to receive either open-label carvedilol or usual care (Fig. 1). A simple stratified 1:1 randomization scheme was planned for those determined to be elevated risk. Therefore, participants were assigned to one of three groups: (1) low risk, nonrandomized; (2) elevated risk, usual care; and (3) elevated risk, carvedilol. After an imbalance was identified with the first 6 elevated risk participants randomized to carvedilol, the protocol was modified to include a stratified block randomization with blocks of four, based on the planned use of trastuzumab. The approach was used to maintain balance across randomized groups.

### Intervention and follow-up

Carvedilol was initiated at a dose of 3.125 mg twice daily on the evening of the first cancer therapy cycle and continued for 12 months. Dose adjustments were based on tolerability and targeted parameters (Supplemental Table 2) as determined by the study physician, and carvedilol was titrated to maximum tolerated dose by the participant or to a SBP of 110–120 mmHg or heart rate of 50–55 beats per minute. Titration steps were predefined at doses of 6.25 mg, 12.5 mg, and up to 25 mg twice daily. Adherence was monitored through pill counts and participant diaries at standardized time intervals (3, 6, 9, and 12 months) for those who randomized to carvedilol group. During the 12 to 24 month period, carvedilol was discontinued unless there was a clinical indication as determined by the study physician. Participants were monitored according to the study procedures described.

### Study procedures

At baseline, and at 3, 6, 9, 12, and 24 months post-cancer treatment initiation, all participants underwent standardized clinical assessment, symptom questionnaires, transthoracic echocardiography, and biomarker evaluation. AEs were also assessed at each of these timepoints using the National Cancer Institute Common Terminology Criteria for Adverse Events (NCI CTCAE) Version 5.0. For the carvedilol group, additional AE assessments occurred during titration visits, and the final AE assessment occurred 30 days following the final dose of study medication.* A priori*, the AEs bradycardia, hypotension, and fatigue were selected for targeted reporting.

### Primary outcome: feasibility, safety, and tolerability

We assessed feasibility through the following measures: (1) the recruitment rate (ratio of consented to approached) and (2) the study withdrawal rate. Safety was ascertained by the incidence of grade 2 or higher AEs as per the NCI CTCAE Version 5.0, including a focused analysis of AEs of interest (bradycardia, hypotension, and fatigue). Carvedilol-related AEs were categorized into five levels of attribution to carvedilol: unrelated, unlikely related, possibly related, probably related, and definitely related. Tolerability was evaluated by (1) the highest carvedilol dose tolerated, (2) the number of carvedilol dose interruptions, (3) adherence to carvedilol, and (4) whether delays or interruptions in cancer therapy were associated with the carvedilol intervention.

### Secondary outcomes: exploratory cardiac efficacy

The cardiac efficacy of carvedilol was explored through quantitative echocardiographic measurements, cardiac biomarkers, and PROs at each study visit over 24 months.

#### Quantitative echocardiography

Quantitative echocardiography was performed at the University of Pennsylvania Center for Quantitative Echocardiography(Philadelphia, PA). All measurements, including 2-dimensional, Doppler, and strain analyses, were performed by a single sonographer, blinded to all participant characteristics, using the TomTec Imaging Systems software. This included quantification of global function (LVEF), cardiac structure (LV mass), diastolic function (E/e’), contractile function (strain [longitudinal, circumferential]), and ventricular-arterial coupling (Ea/ Ees ratio; effective arterial elastance [Ea] and end systolic elastance [Ees]). Left ventricular end-diastolic and end-systolic volumes in the 4-chamber and 2-chamber views were calculated using the Simpson’s method of disks, from which the biplane LVEF was derived. Cardiac dysfunction was defined as an LVEF decline ≥ 10% from baseline to < 50%. Intra-observer coefficients of variation were 7.1% and 12.7% for 4-chamber and 2-chamber LVEF, respectively.

#### Circulating biomarkers

High-sensitivity troponin T (hs-TnT) was quantified using the fourth-generation Elecsys TnT-hs assay on the Cobas platform (Roche Diagnostics) with a range from 3 to 10,000 ng/L. NT-proBNP was measured using the Elecsys NT-proBNP assay on the Cobas platform, with a measuring range of 10–35,000 ng/L. All the biomarker assays were carried out at the Biomarker Discovery and Validation Core of the University of Ottawa Heart Institute.

#### Patient reported outcomes

PROs were assessed with the Functional Assessment of Chronic Illness Therapy (FACIT) fatigue scale for fatigue, the FACIT dyspnea scale short form for dyspnea, and the Godin Leisure-Time Physical Activity Questionnaire for physical activity.

### Sample size estimation

The sample size for this study was calculated based on the minimum number of patients required to detect a statistically significant difference in safety outcomes between the carvedilol and usual care arms. The initial target was to enroll up to 110 patients, stratified into low and elevated risk groups, with an anticipated attrition rate of up to 10%. Specifically, we expected to enroll 56 elevated risk patients, of whom 50 were projected to complete the follow-up. With a sample size of 50 elevated risk and an assumed incidence of hypotension (grade 2 or higher) at 3.1% based upon prior observational data (unpublished), the 95% confidence interval for the incidence rate would have a half-width of 4.8%. For AEs with an assumed incidence of 12%, the target was to estimate a 95% confidence interval with a half-width of 9%. Additionally, with this sample size, the detectable difference in LVEF was 4.9% between the two arms with 80% power and a two-sided alpha of 0.05.

However, substantial enrollment challenges due to the COVID-19 pandemic and decreased use of sequential therapy with anthracyclines and trastuzumab resulted in a lower risk population and fewer enrolled patients than initially planned. Given that only 19 elevated risk participants were ultimately enrolled, the half-width for a 3.1% event rate was estimated to be 7.8%, and for a 12% event rate, this estimate was 14.6%. With this sample size, the detectable difference in LVEF was 8.8% between the two arms with 80% power and a two-sided alpha of 0.05.

### Statistical analysis

Descriptive summary statistics (mean, median, standard deviation, and interquartile range) were used to determine the characteristics of the study population. Feasibility and tolerability were evaluated through an intention-to-treat analysis (“intention-to-treat population”). Safety and exploratory cardiac efficacy were analyzed, evaluating the three groups according to the treatment the participants actually received (“as treated”). For circulating biomarkers, a natural log transformation was applied. Hypothesis testing was not conducted due to the limited sample size and exploratory nature of the study. All analyses were performed using R software (Version 4.3.2, Vienna, Austria).

## Results

### Study population

From August 1, 2019, to July 15, 2022, there were 4553 new patient consults for patients with breast cancer at the Abramson Cancer Center. Of these, there were 576 patients with nonmetastatic breast cancer who planned to initiate either anthracycline or trastuzumab, and of these, 460 patients met all eligibility criteria. Of the eligible patients, 166 were approached and 68 consented to participate and ultimately enrolled in the study. Of these, 49 were classified as low risk and 19 as elevated risk. In the elevated risk group, randomization resulted in 13 participants to the carvedilol intervention and 6 to usual care (Fig. 1, intention-to-treat population). After randomization, three participants refused carvedilol but agreed to remain on study for follow-up, and one participant assigned to usual care started carvedilol for clinical indications at the baseline visit. As a result, there were 11 participants treated with carvedilol and 8 treated as per usual care (Supplemental Fig. 1, as-treated population).

Of the total 68 study participants (median [Q1, Q3] age 52 [42, 61]; 35% Black), 55% had left-sided/bilateral breast cancer, 54% were Stage II (Table 1). Overall, 62% received anthracyclines (median dose of doxorubicin 240 mg/m^2^) with or without trastuzumab, and 40% received left-sided radiation treatment. At baseline, the median (Q1, Q3) LVEF was 61.9% (60.0, 65.0) in the low risk group, 59.6% (57.6, 59.9) in the usual care group, and 61.1% (58.2, 64.8) in the carvedilol group.

### Feasibility, safety, and tolerability

#### Feasibility

The recruitment rate for the study was 41% (68/166) (Table 2). The overall participant withdrawal rate was 7% (5/68), with three withdrawals occurring in the carvedilol and two in the low risk group.

**Table 2:** Feasibility of risk-guided cardioprotection strategy with carvedilol

	Number (%)
Recruitment rate (consented/approached)	68/166 (41%)
*Reasons for declined participation (n = 98*)	
Overwhelmed	38/98 (39%)
Not interested	18/98 (18%)
No reason given	18/98 (18%)
Concerns over carvedilol	11/98 (11%)
Screened fails post-consent	4/98 (4%)
Other^a^	9/98 (9%)
*Study withdrawal rate, Overall*	5/68 (7%)
Low risk, Nonrandomized	2/49 (4%)
Elevated risk, Usual care	0/6 (0%)
Elevated risk, Carvedilol	3/13 (23%)

The study population is presented as intention-to-treat

^a^Refused to be randomized, too much extra time

#### Safety and disease recurrence

In the as-treated study population, targeted AEs of interest (grade 2 or higher) were reported in 36% (4/11) of participants in the carvedilol group, compared to 50% (4/8) in the usual care group, and 51% (25/49) in the low risk group (Table 3). Bradycardia was not observed in any participants, and no cases of hypotension were reported in the carvedilol group. Four participants in the carvedilol group reported fatigue. In one participant, the fatigue was deemed clinically relevant (grade 3) and probably related to carvedilol, leading the participant to reduce the carvedilol dose. In another participant, fatigue (grade 2) was possibly related to carvedilol, while in the remaining two participants (both grade 2), it was considered unlikely to be related to carvedilol. Additional AEs reported in participants randomized to carvedilol included one episode each of Grade 2 presyncope, syncope, and localized edema in three individual participants, although none of these participants experienced hypotension or bradycardia, as per the NCI CTCAE Version 5.0. No serious AEs related to carvedilol were reported. During the median (Q1, Q3) follow-up of 743 (726, 768) days, 6 participants experienced disease recurrence. The distribution of participants according to risk group and intervention were as follows: 5 low risk and 1 elevated risk, assigned to carvedilol. One participant in the low risk group died secondary to COVID-19 infection and adrenal insufficiency. Another participant in the elevated risk group assigned to carvedilol died after cancer progression after the 3-month visit. The 2-year relapse-free survival rate was 92.4%. Details of the grade 2 or higher AEs during the 12-month intervention are described in Supplemental Table 3.

**Table 3:** Safety profile during and after intervention

	Low risk, nonrandomized (*n* = 49)	Elevated risk, usual care (*n* = 8)	Elevated risk, carvedilol (*n* = 11)
During carvedilol intervention (Baseline-12 months)			
Participants experiencing any adverse event			
Grade 2 +	47/49 (96%)	8/8 (100%)	11/11 (100%)
Grade 3 +	15/49 (31%)	2/8 (25%)	6/11 (55%)
Grade 4 +	1/49 (2%)	0/8 (0%)	1/11 (9%)
Grade 5	1/49 (2%)	0/8 (0%)	1/11 (9%)
Participants experiencing any targeted adverse event of interest (bradycardia, fatigue, hypotension)			
Grade 2 +	25/49 (51%)	4/8 (50%)	4/11 (36%)
Grade 3 +	2/49 (4%)	1/8 (13%)	1/11 (9%)
Grade 4 +	1/49 (2%)	0/8 (0%)	0/11 (0%)
Grade 5	0/49 (0%)	0/8 (0%)	0/11 (0%)
Post carvedilol intervention (12 ~ 24 months)			
Participants experiencing any adverse event			
Grade 2 +	12/47 (26%)	3/8 (38%)	0/8 (0%)
Grade 3 +	2/47 (4%)	0/8 (0%)	0/8 (0%)
Grade 4 +	1/47 (2%)	0/8 (0%)	0/8 (0%)
Grade 5	0/47 (0%)	0/8 (0%)	0/8 (0%)
Participants experiencing any targeted adverse event of interest (bradycardia, fatigue, hypotension)			
Grade 2 +	0/47 (0%)	1/8 (13%)	0/8 (0%)
Grade 3 +	0/47 (0%)	0/8 (0%)	0/8 (0%)
Grade 4 +	0/47 (0%)	0/8 (0%)	0/8 (0%)
Grade 5	0/47 (0%)	0/8 (0%)	0/8 (0%)

The study population is presented as treated, with data shown as number (%)

#### Tolerability

In the intention-to-treat population of the carvedilol group, carvedilol was titrated to the maximum dose of 25 mg twice daily in one out of 13 participants, while the median maximum dose was 6.25 mg twice daily (Table 4). The median (Q1, Q3) of medication adherence monitored via pill counts was 93% (14%, 97%).

**Table 4:** Tolerability of risk-guided cardioprotection strategy with carvedilol

	Low risk, nonrandomized (*n* = 49)	Elevated risk, usual care (*n* = 6)	Elevated risk, carvedilol (*n* = 13)
Completion of 12-month study visits	47/49 (96%)	6/6 (100%)	10/13 (77%)
Completion of 12-month carvedilol intervention	N/A	N/A	7/13 (54%)
Maximally tolerated dose achieved, Median	N/A	N/A	6.25 mg twice daily
3.125 mg twice daily	N/A	N/A	2/7 (29%)
6.25 mg twice daily	N/A	N/A	4/7 (57%)
12.5 mg twice daily	N/A	N/A	0/7 (0%)
25 mg twice daily	N/A	N/A	1/7 (14%)
Carvedilol dose interruption	N/A	N/A	1/7 (14%)
Adherence to carvedilol intervention, Median^a^	N/A	N/A	93%
Mean	N/A	N/A	64%
Premature carvedilol termination	N/A	N/A	6/13 (46%)
Refused to start	N/A	N/A	3/6 (50%)
3.125 mg twice daily	N/A	N/A	3/6 (50%)
Chemotherapy interruption	14/49 (29%)	2/6 (33%)	4/13 (31%)
Due to cardiac dysfunction	0/14 (0%)	0/2 (0%)	0/4 (0%)
Due to carvedilol intervention	N/A	N/A	0/4 (0%)

The study population is presented as intention-to-treat, with data shown as median (Q1, Q3) or number (%). *N/A* not applicable

^a^via pill count

However, of the participants assigned to carvedilol, three declined but continued their participation in study visits undergoing echocardiograms, biomarkers, and questionnaires. The reasons were all related to concerns of potential AEs. As a result, in the as-treated population of 10 participants who were randomized to and agreed to take carvedilol, the median (Q1, Q3) adherence rate via pill counts was 94% (91%, 98%). Over time, among these 10 participants, three withdrew from the study for reasons unrelated to carvedilol, including feeling overwhelmed secondary to breast cancer, COVID-19, and disease progression.

Across the intention-to-treat population, chemotherapy interruptions were reported in 29% of the low risk group, 33% in the usual care group, and 31% in the carvedilol group. None of these interruptions were related to cardiac dysfunction or carvedilol.

### Exploratory cardiac efficacy

During follow-up, only one (1.5%) participant experienced cardiac dysfunction (60.9% at baseline to 49.3% at 9 months and 48.6% at 12 months) but recovered at 24 months to 63.9% without any medications or other intervention. This participant was initially classified as low risk, received anthracyclines but not trastuzumab, and did not undergo radiation treatment.

**Fig. 2:**
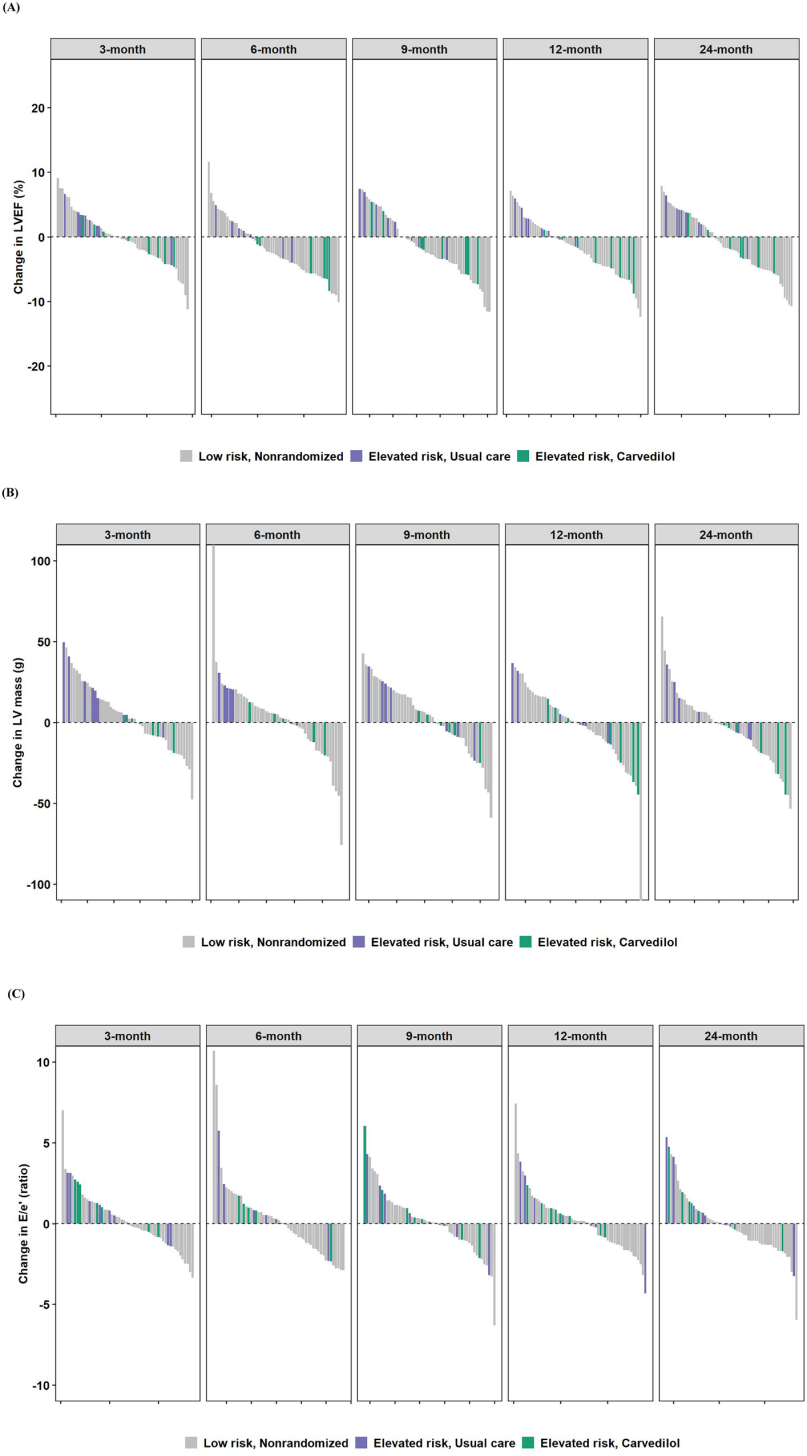

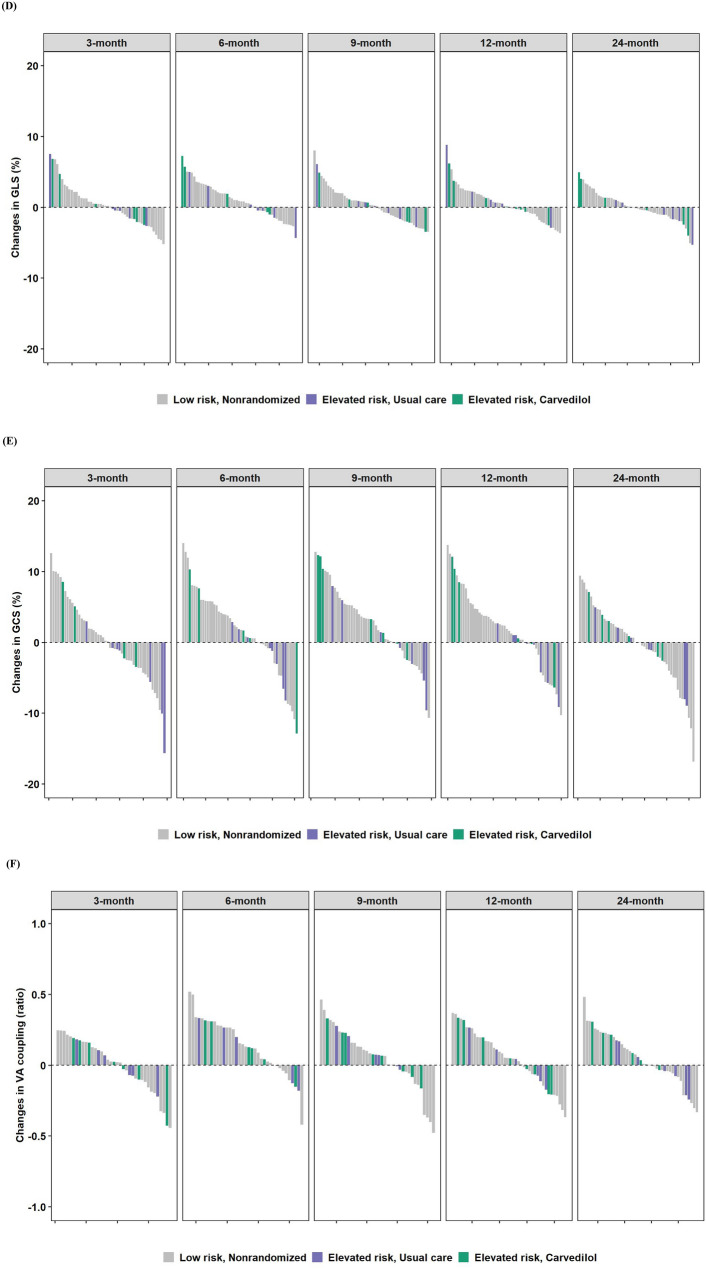

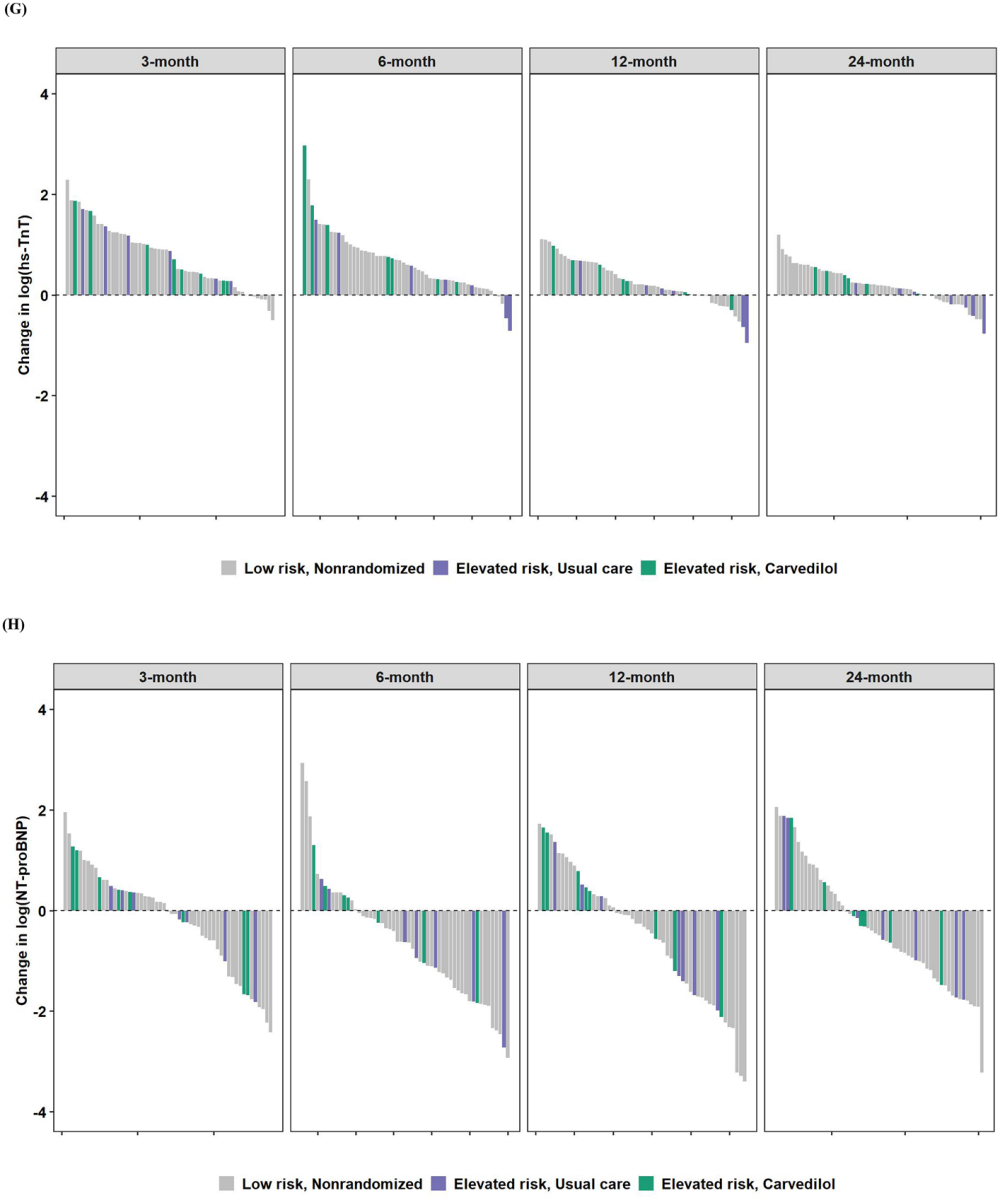
Changes from baseline echocardiographic parameters and circulating biomarkers. **A** LVEF (%), **B** LV mass (g), **C** E/e’ (ratio), **D** GLS (%), **E** GCS (%), **F** VA coupling (ratio), **G** hs-TnT, **H** NT-proBNP. Study groups are represented by different colors. Low risk, Nonrandomized (Gray); Elevated risk, Usual care (Purple), Elevated risk, Carvedilol (Green). For changes in biomarkers (**G**, **H**), a natural log transformation was applied. A unit increment from baseline should be interpreted as a doubling

Across all as-treated groups, the median values for LVEF and GLS at 24 months appeared similar (Supplemental Table 4). Interestingly, LV mass tended to decrease in those in the elevated risk subgroup treated with carvedilol. During the first six months of breast cancer treatment, the median change in LV mass was −4.6 g (−8.7 g, 1.4 g) at 3 months, and this further decreased at 12 and 24 months to lower than baseline values (Fig. 2B). In contrast, in the elevated risk, usual care and in the low risk groups, this same pattern was not evident, as there were no substantial differences from baseline LV mass at 12 months. There were no clinically meaningful differences in other echocardiographic parameters.

There were low-grade elevations in hs-TnT across all as-treated groups; (Fig. 2G, Supplemental Table 4). A great deal of variability was noted particularly with NT-proBNP, and in general, higher values were noted with those in the elevated risk subgroup treated with carvedilol, even at baseline. Decreases in NT-proBNP tended to occur over time, particularly at 24 months (Fig. 2H, Supplemental Table 4).

At 12 months, the PROs in the carvedilol group were generally favorable (Supplemental Table 5). These effects were attenuated at 24 months, a time period reflective of participants being off carvedilol.

## Discussion

In this Phase 1 randomized clinical trial, risk-guided carvedilol appeared to be generally feasible, safe, and tolerable during breast cancer treatment at a maximum dose of 6.25 mg twice daily. Although hypothesis testing was not performed secondary to the limited sample size, there was no strong indication of clinically important differences in echocardiographic or biomarker measures during the first six months. However, those treated with carvedilol tended to experience a decrease in LV mass. In addition, PROs in the carvedilol group were qualitatively favorable at 12 months.

The fundamental question of our study was to assess the feasibility of risk-guided cardioprotection using carvedilol. Our Phase 1 results provide some evidence to support the feasibility of this strategy with a high number of eligible patients, strong recruitment rates, and high medication adherence among those who agreed to initiate carvedilol. On the other hand, our study also highlights the significant challenges of introducing a non-treatment intervention trial at the time of cancer therapy initiation, particularly during a global pandemic. Three participants declined to start carvedilol, underscoring challenges in acceptance. All three participants had low SBPs at baseline (in the 100 mmHg range) which were the lowest among all study participants but still higher than the threshold for exclusion from this study (90 mmHg). They did not have a previous history of hypertension, diabetes, hyperlipidemia, or cardiovascular disease. This may have influenced their perception of any potential benefits of cardiac medications. Overall, five participants did not complete the study secondary to death or withdrawal.

Although making inferences from the small number of participants should be approached with caution, we feel our study emphasizes the critical need to define both the efficacy and patient acceptability of cardiac interventions, particularly as it pertains to the optimal timing of cardioprotection. As the time of breast cancer diagnosis and active cancer treatment is often very overwhelming, introducing cardioprotection strategies immediately at the time of cancer therapy completion, assuming no compromise in efficacy, could be a more acceptable strategy. Our study highlights the need for continued iteration and development of optimized strategies to enhance the design and execution of multi-disciplinary, non-treatment trials in our participants with cancer who are at risk for non-cancer comorbidities.

Evaluation of our exploratory echocardiographic parameters of cardiac structure, function, and remodeling raises hypotheses that carvedilol may mitigate adverse cardiac remodeling through an attenuation of potential early increases in LV mass with breast cancer treatment. This result is consistent with the efficacy of carvedilol in reducing LV mass in heart failure participants [9, 13], and the patterns in LV mass for both the usual care and low risk groups align with those observed in previous studies [14]. While we were unable to determine an effect of carvedilol on subclinical measures of cardiotoxicity (biomarkers, echocardiography measures) or the rate of cardiac dysfunction secondary to our very small sample size, these findings raise important hypotheses related to potential effects of carvedilol on cardiac hypertrophy and ventricular remodeling. In addition, participants treated with carvedilol had generally favorable PROs over the 12-month intervention, suggesting possible additional benefits.

The distinctive feature of our trial was the use of a cardiotoxicity risk score to select patients who may derive greater benefit from cardioprotection. This diverges from traditional cardio-oncology trials, which have historically relied on echocardiographic parameters [15] or cardiac biomarkers [16, 17]. However, we acknowledge the need for advances in robust clinical risk prediction algorithms; our score requires external validation prior to broader clinical application [18–20].

The limitations of our study include its small sample size, which constrained hypothesis testing. The concomitant use of cardiac medications such as ACE inhibitors/ARBs or statins was not controlled (Supplemental Table 6), which could impact our exploratory efficacy outcome. Lastly, the execution of the study during the COVID-19 pandemic impacted patient recruitment and adherence.

## Conclusion

This Phase 1 trial provides evidence that risk-guided cardioprotection with carvedilol at a dose of 6.25 mg twice daily is generally feasible, safe, and well tolerated during breast cancer treatment in a racially diverse population . However, additional strategies are necessary to optimize the study design and execution of non-treatment trial interventions at the initiation of cancer therapy.

## Supplementary Information

Below is the link to the electronic supplementary material.

Supplementary file1 (DOCX 464 KB)

## Data Availability

Data is provided within the manuscript or supplementary information files. The datasets used and/or analyzed during the current study are available from the corresponding author on reasonable request.

## Abbreviations

AE: Adverse event
DBP: Diastolic blood pressure
GCS: Global circumferential strain
GLS: Global longitudinal strain
hs-TnT: High-sensitivity troponin T
LVEF: Left ventricular ejection fraction
NT-proBNP: N-terminal pro b-type natriuretic peptide
PRO: Patient-reported outcome
SBP: Systolic blood pressure
