# Supplementary material for: Risk-guided cardioprotection with carvedilol in patients with breast cancer (CCT guide): a phase 1 randomized clinical trial
Source: Breast Cancer Res Treat. 2025 Apr 2;211(2):293–305. doi: 10.1007/s10549-025-07636-3 (PMC12006281; doi:10.1007/s10549-025-07636-3)

**Supplementary Content**

**Supplemental Method** Derivation and validation of a cardiotoxicity risk score

**Supplemental Table 1** Eligibility criteria of study population

**Supplemental Table 2** Targeted parameters for carvedilol up-titration

**Supplemental Table 3** Number of participants who experienced at least one grade 2 or higher adverse event during the intervention (Baseline - 12 month)

**Supplemental Table 4** Descriptive statistics of quantitative echocardiographic parameters and circulating biomarkers

**Supplemental Table 5** Patient-reported outcomes

**Supplemental Table 6** Changes in taking ACEi/ARB and statin

**Supplemental Figure 1** CONSORT diagram (as treated population)

**Supplemental Method** Derivation and validation of a cardiotoxicity risk score

We developed and internally validated a cardiotoxicity risk score aimed at predicting declines in left ventricular ejection fraction (LVEF) among patients undergoing cancer therapy with doxorubicin and/or trastuzumab. The study was conducted under the Penn Cardiotoxicity of Cancer Therapy (CCT, NCT01173341), a prospective cohort study at the University of Pennsylvania. Enrollment included participants aged 18 years or older, diagnosed with breast cancer, and treated from 2011 to 2018. All participants provided written informed consent. Clinical profiles, biomarkers, and echocardiographic data were systematically collected at baseline and periodically thereafter. Cardiotoxicity was defined specifically as a reduction in LVEF by ≥10% to below 50% following the initiation of therapy. Characteristics of variables among the total population and coefficients in the risk model are shown below.

| Characteristic | Total population  (N=611) | Coefficient in risk model  (log hazard ratio scale) |
| --- | --- | --- |
| Age (years) | 50.1 ± 11.5 |  |
| Sex |  |  |
| Female | 611 (100) |  |
| Race |  |  |
| White | 417 (68.2) | -0.06 |
| African American | 153 (25.0) | Reference |
| Other | 41 (6.7) | 0.47 |
| Treatment regimen |  |  |
| Doxorubicin | 364 (59.6) | Reference |
| Trastuzumab | 159 (26.0) | -0.76 |
| Doxorubicin+Trastuzumab | 88 (14.4) | 1.20 |
| Pertuzumab | 73 (11.9) |  |
| Radiotherapy | 381 (65.4) |  |
| Hypertension | 184 (30.3) | 0.10 |
| Hyperlipidemia | 146 (24.1) |  |
| Smoking, prior or current | 232 (38.7) | 0.13 |
| Diabetes | 55 (9.1) | 0.53 |
| Systolic Blood Pressure (mmHg) | 126.5 ± 16.6 | 0.01 |
| Body Mass Index (kg/m^2^) | 28.0 ± 6.6 |  |
| Baseline Clinical LVEF | 62.3 ± 5.2 | -0.10 |

Number (%) or Mean ± standard deviation are presented. LVEF, left ventricular ejection fraction.

**Supplemental Table 1** Eligibility criteria of study population

| Inclusion criteria |
| --- |
| 1. Females  2. ≥ 18 years old  3. Diagnosed with breast cancer, with treatment plan to include therapy with anthracyclines and/or trastuzumab/trastuzmab-anns in the adjuvant or neoadjuvant setting  4. Able to swallow tablets  5. Study team is able to obtain all necessary information for calculating baseline cardiotoxicity risk (including echocardiographic images for quantitation of LVEF) prior to enrollment  6. Standard of care pre-chemotherapy measurement of LVEF by echocardiogram |
| Exclusion criteria |
| 1. Known Stage IV breast cancer at enrollment  2. Pregnant or breast feeding. Due to unknown risks and potential harm to the unborn fetus a negative pregnancy test (serum or urine) within 10 days prior to enrollment is required in women with child-bearing potential. Due to the potential nursing infant harm, women who are currently breast feeding are not eligible for this study.  3. Contraindication to Carvedilol  3-A. Baseline systolic blood pressure < 90mmHg (if multiple blood pressures were available in the medical record within 1 month prior to screening, the average SBP was be considered)  3-B. Baseline heart rate < 55 bpm consistent with severe bradycardia (if multiple resting heart rates were available in the medical record within 1 month prior to screening, the average heart rate was be considered)  3-C. Allergy to Carvedilol  3-D. History of bronchial asthma or related bronchospastic conditions. If asthma is listed in the medical history or problem list in the EMR but the patient reports no current asthma diagnosis and there are no medical visits with a diagnosis of asthma in the preceding 12 months, the patient was considered not to have asthma and was not excluded.  3-E. Known history of sick sinus syndrome  3-F. Severe hepatic impairment, defined as serum bilirubin > 3.0x UNL, AST or ALT > 5.0 ULN within 28 days of enrollment  3-G. Second- or third-degree AV block, as determined by electrocardiogram  3-H. Severe bradycardia (unless permanent pacemaker is in place)  3-I. Patients in cardiogenic shock or decompensated heart failure requiring the use of IV inotropic therapy  3-J. Current use of Bupropion (Wellbutrin^®^), Fluoxetine (Prozac^®^), Paroxetine (Paxil^®^), Quinidine (Quinidex^®^), Duloxetine (Cymbalta^®^), Digoxin  4. Current treatment with beta blocker  5. Unable to provide consent |

LVEF, left ventricular ejection fraction; EMR, electronic medical record; UNL, upper normal limit; AST, Aspartate Aminotransferase; ALT, Alanine Aminotransferase; AV, Atrioventricular block; IV, intravenous.

**Supplemental Table 2** Targeted parameters for carvedilol up-titration

| The dose was increased to the next level, taken twice daily, if all the following conditions were met:   1. Systolic Blood Pressure (SBP) > 110mmHg (if multiple blood pressures available since last visit, average SBP will be considered); 2. Resting heart rate (HR) > 50bpm (if multiple heart rates available since last visit, average HR will be considered); 3. No adverse events (AEs)/serious adverse events (SAEs) which are possibly, probably, or definitely related to the intervention are reported; 4. Compliance between 80% and 120%   If one or more of the above conditions is not met, the PI will determine whether to continue at current dose, titrate to next higher dose level, or titrate to next lower dose level. |
| --- |

**Supplemental Table 3** Number of participants who experienced at least one grade 2 or higher targeted adverse event during the intervention (Baseline - 12 months)

|  | Low risk, Nonrandomized  (n=49) | Elevated risk,  Usual care  (n=8) | Elevated risk,  Carvedilol  (n=11) |
| --- | --- | --- | --- |
| Fatigue | 23 (47%) | 4 (50%) | 4 (36%) |
| Nausea | 9 (18%) | 1 (13%) | 3 (27%) |
| Arthralgia | 12 (24%) | 1 (13%) | 2 (18%) |
| Weight Gain | 6 (12%) | 1 (13%) | 1 (9%) |
| Hypotension | 3 (6%) | 0 | 0 |
| Dizziness | 4 (8%) | 0 | 0 |
| Pre-syncope | 1 (2%) | 1 (13%) | 1 (9%) |
| Syncope | 2 (4%) | 0 | 1 (9%) |
| Hyperglycemia | 0 | 1 (13%) | 1 (9%) |
| Vomiting | 4 (8%) | 0 | 1 (9%) |
| Headache | 4 (8%) | 0 | 1 (9%) |
| Heart Failure | 1 (2%) | 0 | 0 |
| Cough | 2 (4%) | 1 (13%) | 1 (9%) |
| Localized Edema | 2 (4%) | 0 | 0 |
| Edema, Limbs Localized Edema | 0 | 0 | 1 (9%) |
| Lymphedema | 2 (4%) | 0 | 0 |
| Disease progression | 0 | 0 | 1 (9%) |
| Cardiac arrest (related to COVID-19) | 1 (2%) | 0 | 0 |
| Bradycardia | 0 | 0 | 0 |

The study population is presented as treated, with data shown as number (%).

**Supplemental Table 4** Descriptive statistics of quantitative echocardiographic parameters and circulating biomarkers

|  | | Low risk, Nonrandomized  (n=49) | Elevated risk,  Usual care  (n=8) | Elevated risk,  Carvedilol  (n=11) |
| --- | --- | --- | --- | --- |
| **LVEF ^a^, (%)** | Baseline | 61.9 (60.0, 65.0) | 59.0 (57.2, 60.4) | 61.1 (59.7, 64.9) |
| 3-month | | 60.9 (58.8, 62.8) | 61.1 (58.8, 62.9) | 60.1 (59.0, 62.3) |
| 6-month | | 59.7 (57.2, 62.0) | 58.5 (56.7, 60.9) | 58.8 (58.1, 59.7) |
| 9-month | | 60.4 (58.2, 63.1) | 62.6 (57.3, 65.7) | 59.3 (57.8, 64.8) |
| 12-month | | 59.7 (56.9, 62.3) | 60.4 (59.1, 61.9) | 58.4 (57.4, 59.3) |
| 24-month | | 60.3 (58.0, 62.6) | 61.7 (59.4, 62.5) | 61.7 (57.3, 62.3) |
| **LV mass, g** | Baseline | 98.0 (84.5, 120.0) | 95.3 (87.2, 103.6) | 110.6 (87.0, 124.9) |
| 3-month | | 106.1 (93.2, 117.1) | 124.0 (114.3, 125.3) | 101.3 (87.1, 110.1) |
| 6-month | | 100.6 (84.6, 121.6) | 110.0 (92.6, 122.8) | 108.3 (77.3, 117.5) |
| 9-month | | 100.0 (84.8, 114.7) | 111.0 (86.2, 133.7) | 93.9 (74.9, 113.7) |
| 12-month | | 94.5 (87.2, 112.2) | 98.4 (90.2, 138.0) | 92.2 (75.4, 103.6) |
| 24-month | | 97.8 (90.0, 108.5) | 96.9 (85.5, 120.8) | 80.2 (65.6, 91.9) |
| **E/e’ (ratio)** | Baseline | 7.64 (6.54, 8.70) | 6.85 (6.07, 7.78) | 7.86 (6.89, 8.60) |
| 3-month | | 7.28 (5.81, 8.38) | 7.74 (7.06, 9.00) | 8.69 (7.98, 9.60) |
| 6-month | | 7.37 (5.89, 9.43) | 7.31 (6.87, 9.84) | 8.48 (6.99, 8.65) |
| 9-month | | 7.18 (6.22, 8.51) | 7.33 (6.63, 9.40) | 7.76 (6.22, 8.49) |
| 12-month | | 7.06 (6.46, 8.45) | 8.24 (6.79, 8.82) | 8.22 (7.65, 9.18) |
| 24-month | | 7.04 (6.43, 7.99) | 7.90 (7.20, 8.68) | 8.25 (7.05, 8.67) |
| **GLS, (%)** | Baseline | -21.8 (-22.9, -20.6) | -20.5 (-23.0, -20.1) | -20.7 (-21.8, -20.2) |
| 3-month | | -21.8 (-23.2, -20.2) | -21.3 (-21.5, -20.7) | -21.9 (-22.1, -21.3) |
| 6-month | | -20.7 (-22.1, -19.9) | -20.9 (-23.0, -20.4) | -20.0 (-21.2, -19.9) |
| 9-month | | -21.4 (-23.1, -19.9) | -21.3 (-22.3, -19.7) | -21.5 (-23.3, -20.3) |
| 12-month | | -21.3 (-23.2, -19.8) | -20.0 (-21.6, -19.4) | -21.1 (-22.6, -20.0) |
| 24-month | | -21.3 (-22.6, -20.0) | -22.1 (-25.3, -20.2) | -22.0 (-22.9, -20.3) |
| **GCS, (%)** | Baseline | -27.9 (-30.6, -23.4) | -24.4 (-27.5, -22.7) | -29.0 (-34.4, -24.3) |
| 3-month | | -26.5 (-29.7, -22.7) | -29.9 (-30.3, -24.9) | -26.9 (-29.6, -24.4) |
| 6-month | | -24.0 (-27.1, -22.3) | -26.2 (-27.2, -25.4) | -26.5 (-27.9, -24.3) |
| 9-month | | -25.2 (-28.3, -21.7) | -25.5 (-28.4, -23.1) | -23.1 (-24.3, -22.4) |
| 12-month | | -25.2 (-28.2, -21.9) | -26.2 (-30.1, -23.6) | -24.3 (-25.3, -22.9) |
| 24-month | | -27.2 (-31.1, -23.3) | -24.7 (-29.7, -22.3) | -25.0 (-27.9, -22.5) |
| **Ea/Ees ratio** | Baseline | 0.71 (0.58, 0.92) | 0.84 (0.68, 0.95) | 0.76 (0.62, 0.83) |
| 3-month | | 0.76 (0.69, 0.84) | 0.79 (0.68, 0.90) | 0.82 (0.66, 0.94) |
| 6-month | | 0.89 (0.73, 0.99) | 0.83 (0.79, 0.86) | 0.87 (0.71, 1.05) |
| 9-month | | 0.78 (0.71, 0.97) | 0.88 (0.77, 0.97) | 0.90 (0.66, 1.06) |
| 12-month | | 0.82 (0.71, 0.93) | 0.88 (0.84, 0.89) | 0.83 (0.64, 0.94) |
| 24-month | | 0.82 (0.71, 0.96) | 0.78 (0.74, 0.89) | 0.83 (0.78, 0.94) |
| **hs-TnT, ng/L** | Baseline | 5.7 (4.1, 8.0) | 8.0 (6.4, 8.4) | 6.1 (4.7, 7.3) |
| 3-month | | 9.1 (7.1, 16.0) | 11.9 (9.8, 16.0) | 11.6 (8.6, 15.2) |
| 6-month | | 8.5 (6.6, 12.3) | 7.2 (5.7, 14.5) | 13.0 (10.2, 15.3) |
| 12-month | | 7.2 (5.6, 9.4) | 6.2 (4.2, 8.7) | 7.4 (6.4, 8.0) |
| 24-month | | 6.4 (5.3, 8.2) | 6.0 (4.7, 7.3) | 7.0 (5.6, 8.0) |
| **NT-proBNP, ng/L** | Baseline | 98.4 (35.5, 161.6) | 54.8 (43.2, 74.3) | 88.4 (57.1, 100.1) |
| 3-month | | 63.4 (30.0, 140.5) | 49.7 (41.9, 66.0) | 111.6 (84.1, 118.9) |
| 6-month | | 43.1 (28.9, 93.7) | 33.3 (24.6, 39.9) | 105.1 (50.9, 115.9) |
| 12-month | | 52.0 (25.3, 108.9) | 32.6 (19.6, 82.7) | 126.8 (23.2, 143.9) |
| 24-month | | 46.6 (30.1, 113.5) | 53.9 (33.6, 91.6) | 81.7 (54.9, 110.5) |

The study population is presented as treated, with data shown as median (Q1, Q3) are presented. LVEF, left ventricular ejection fraction; GLS, global longitudinal strain; GCS, global circumferential strain. LV mass was calculated by area-length method.

**Supplemental Table 5** Patient-reported outcomes

|  | | Low risk, Nonrandomized  (n=49) | Elevated risk,  Usual care  (n=8) | Elevated risk,  Carvedilol  (n=11) |
| --- | --- | --- | --- | --- |
| **FACIT fatigue scale^1^** | Baseline | 48 (34, 52) | 48 (43, 49) | 52 (43, 53) |
| 3-month | | 26 (19, 39) | 32 (24, 37) | 36 (18, 46) |
| 6-month | | 44 (34, 50) | 36 (23, 43) | 42 (28, 48) |
| 9-month | | 44 (35, 47) | 46 (40, 50) | 48 (34, 50) |
| 12-month | | 46 (34, 51) | 43 (34, 47) | 50 (42, 52) |
| 24-month | | 45 (34, 52) | 47 (42, 48) | 45 (39, 46) |
| **FACIT dyspnea scale short form^2^** | Baseline | 1 (0, 7) | 4 (3, 6) | 0 (0, 0) |
| 3-month | | 7 (0, 11) | 6 (4, 8) | 5 (2, 8) |
| 6-month | | 3 (1, 9) | 7 (2, 10) | 3 (1, 11) |
| 9-month | | 2 (0, 8) | 0 (0, 2) | 2 (0, 3) |
| 12-month | | 2 (0, 6) | 0 (0, 2) | 1 (0, 2) |
| 24-month | | 1 (0, 6) | 2 (0, 3) | 3 (0, 5) |
| **Godin total score^3^** | Baseline | 28 (9, 56) | 10 (2, 19) | 24 (10, 29) |
| 3-month | | 29 (6, 56) | 2 (0, 16) | 3 (0, 30) |
| 6-month | | 26 (11, 41) | 15 (3, 28) | 19 (8, 36) |
| 9-month | | 32 (18, 59) | 27 (11, 40) | 41 (5, 46) |
| 12-month | | 40 (18, 58) | 32 (28, 44) | 36 (13, 79) |
| 24-month | | 33 (16, 58) | 14 (2, 65) | 9 (5, 49)^*^ |
| **Godin MVPA score^3^** | Baseline | 19 (0, 47) | 0 (0, 0) | 11 (0, 16) |
| 3-month | | 3 (0, 35) | 0 (0, 7) | 0 (0, 15) |
| 6-month | | 10 (0, 25) | 0 (0, 19) | 10 (0, 25) |
| 9-month | | 19 (9, 43) | 5 (0, 29) | 30 (0, 33) |
| 12-month | | 28 (8, 47) | 16 (10, 30) | 22 (5, 59) |
| 24-month | | 24 (5, 43) | 3 (0, 49) | 0 (0, 31)^*^ |

The study population is presented as treated, with data shown as medians (Q1, Q3). FACIT, Functional Assessment of Chronic Illness Therapy; MVPA, Moderate-Vigorous Physical Activity

^1^ the higher the score, the better the quality of life

^2^ the higher the score, the worse the dyspnea

^3^ The higher the score, the more activity.

^*^ Note: Affected by notable declines in scores for two participants who either had no data or recorded zero scores by the 24-month follow-up.

**Supplemental Table 6** Changes in taking ACEi/ARB and statin

|  | **Baseline** | **3-month** | **6-month** | **9-month** | **12-month** | **24-month** |
| --- | --- | --- | --- | --- | --- | --- |
| **Number of subjects taking ACEi/ARB** |  |  |  |  |  |  |
| Low risk, Nonrandomized (n=49) | 6/49 (12%) | 5/49 (10%) | 6/48 (13%) | 6/48 (13%) | 6/47 (13%) | 6/47 (13%) |
| Elevated risk, Usual care (n=6) | 1/6 (17%) | 1/6 (17%) | 1/6 (17%) | 1/6 (17%) | 1/6 (17%) | 2/6 (33%) |
| Elevated risk, Carvedilol (n=13) | 1/13 (8%) | 0/12 (0%) | 2/10 (20%) | 2/10 (20%) | 1/10 (10%) | 2/10 (20%) |
| **Number of subjects taking statin** |  |  |  |  |  |  |
| Low risk, Nonrandomized (n=49) | 6/49 (12%) | 6/49 (12%) | 6/48 (13%) | 6/48 (13%) | 5/47 (11%) | 10/47 (21%) |
| Elevated risk, Usual care (n=6) | 1/6 (17%) | 1/6 (17%) | 2/6 (33%) | 2/6 (33%) | 2/6 (33%) | 2/6 (33%) |
| Elevated risk, Carvedilol (n=13) | 1/13 (8%) | 1/12 (8%) | 2/10 (20%) | 3/10 (30%) | 2/10 (20%) | 2/10 (20%) |

The study population is presented as intention-to-treat, with data shown as number and percentages (%). ACEi, Angiotensin-Converting Enzyme Inhibitors; ARB, Angiotensin II Receptor Blockers.

**Supplemental Figure 1** CONSORT diagram (as treated population)


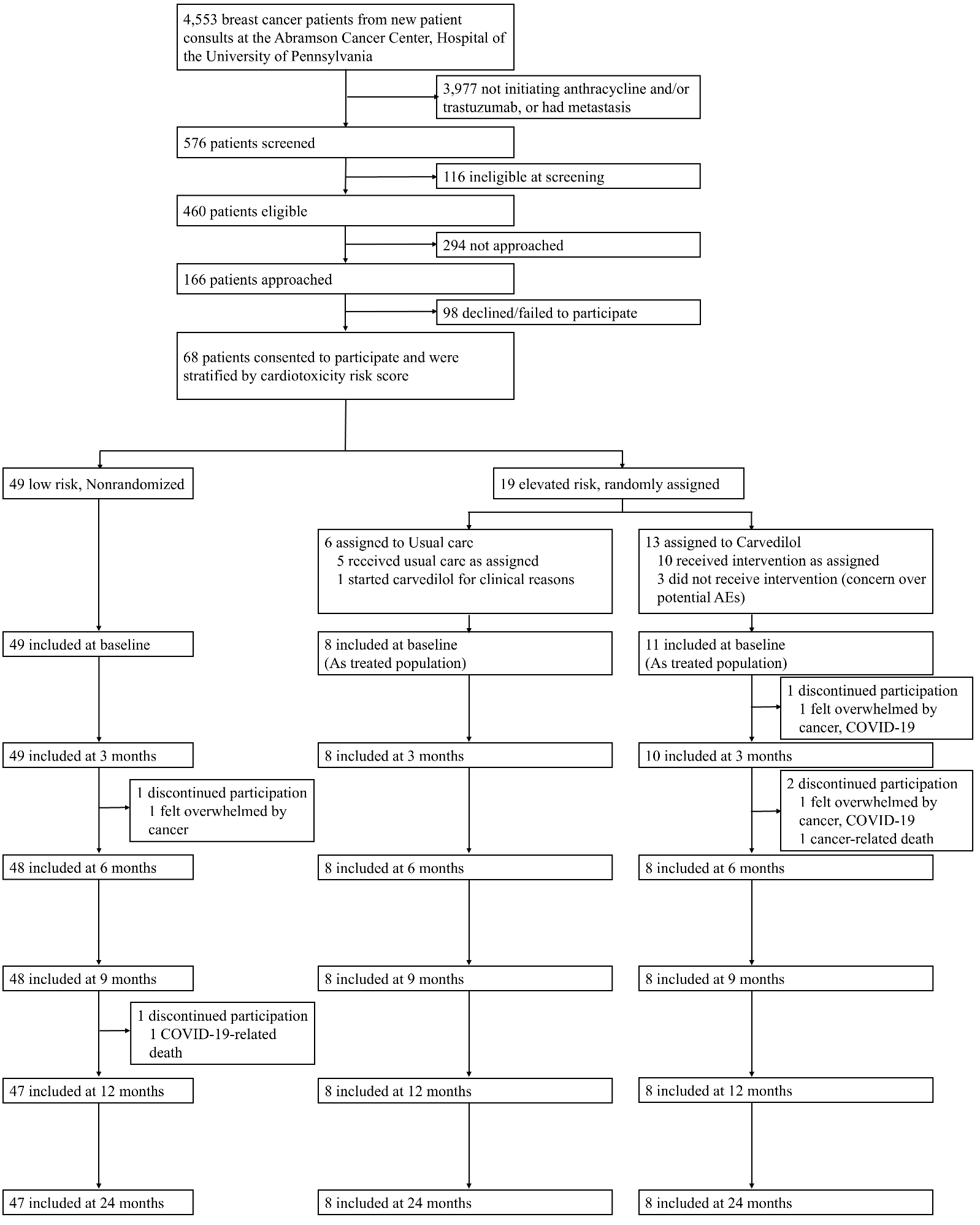

Supplement: Supplementary file 1 — Supplementary file1 (DOCX 464 KB) [file 10549_2025_7636_MOESM1_ESM.docx]
